# Histone Acetyltransferase-Dependent Pathways Mediate Upregulation of NADPH Oxidase 5 in Human Macrophages under Inflammatory Conditions: A Potential Mechanism of Reactive Oxygen Species Overproduction in Atherosclerosis

**DOI:** 10.1155/2019/3201062

**Published:** 2019-09-02

**Authors:** Mihaela-Loredana Vlad, Simona-Adriana Manea, Alexandra-Gela Lazar, Monica Raicu, Horia Muresian, Maya Simionescu, Adrian Manea

**Affiliations:** ^1^Institute of Cellular Biology and Pathology “Nicolae Simionescu” of the Romanian Academy, Bucharest, Romania; ^2^University Hospital Bucharest, Cardiovascular Surgery Department, Bucharest, Romania

## Abstract

Histone acetylation plays a major role in epigenetic regulation of gene expression. Monocyte-derived macrophages express functional NADPH oxidase 5 (Nox5) that contributes to oxidative stress in atherogenesis. The mechanisms of Nox5 regulation are not entirely elucidated. The aim of this study was to investigate the expression pattern of key histone acetyltransferase subtypes (p300, HAT1) in human atherosclerosis and to determine their role in mediating the upregulation of Nox5 in macrophages under inflammatory conditions. Human nonatherosclerotic and atherosclerotic tissue samples were collected in order to determine the expression of p300 and HAT1 isoforms, H3K27ac, and Nox5. In vitro determinations were done on human macrophages exposed to lipopolysaccharide in the absence or presence of histone acetyltransferase inhibitors. Western blot, immunohistochemistry, immunofluorescence, real-time PCR, transfection, and chromatin immunoprecipitation assay were employed. The protein levels of p300 and HAT1 isoforms, H3K27ac, and Nox5 were found significantly elevated in human atherosclerotic specimens. Immunohistochemistry/immunofluorescence staining revealed that p300, HAT1, H3K27ac, H3K9ac, and Nox5 proteins were colocalized in the area of CD45^+^/CD68^+^ immune cells and lipid-rich deposits within human atherosclerotic plaques. Lipopolysaccharide induced the levels of HAT1, H3K27ac, H3K9ac, and Nox5 and the recruitment of p300 and HAT1 at the sites of active transcription within Nox5 gene promoter in cultured human macrophages. Pharmacological inhibition of histone acetyltransferase significantly reduced the Nox5 gene and protein expression in lipopolysaccharide-challenged macrophages. The overexpression of p300 or HAT1 enhanced the Nox5 gene promoter activity. The histone acetyltransferase system is altered in human atherosclerosis. Under inflammatory conditions, HAT subtypes control Nox5 overexpression in cultured human macrophages. The data suggest the existence of a new epigenetic mechanism underlying oxidative stress in atherosclerosis.

## 1. Introduction

Studies on human and experimental models of atherosclerosis provide compelling evidence that oxidative stress plays a major role in all stages of atheroma formation [1–3]. Produced in excess, reactive oxygen species (ROS) induce structural and functional alterations of biological molecules and trigger redox-sensitive proinflammatory signalling pathways by acting on specific protein kinases/phosphates and transcription factors [4]. Thus, counteracting ROS overproduction by pharmacologically targeting the enzymatic sources of ROS and/or their upstream regulators may lead to the attenuation of atheroma formation.

Among various cell types implicated in the atherogenesis, macrophages (Mac) are the major sources of ROS and oxidative stress. Reportedly, members of the NADPH oxidase (Nox) family are the main contributors to ROS production in Mac. Whilst Nox1, Nox2, and Nox4 type oxidases have been extensively investigated in various experimental models [5–8], the precise function and the regulatory mechanisms of the newly identified calcium-dependent Nox5 in Mac remain elusive, especially in atherosclerosis [9, 10]. Previously, we have reported that several proinflammatory transcription factors such as nuclear factor kB (NF-*κ*B), activator protein-1 (AP-1), signal transducers and activation of transcription 1/3 (STAT1/3), and CCAAT-enhancer-binding proteins *α*/*β*/*δ* (C/EBP*α*/*β*/*δ*) are coordinately involved in the regulation of Nox5 expression via direct/indirect mechanisms [11, 12]. Yet, the functions of the aforementioned transcription factors are critically modulated by epigenetic changes in chromatin topology [13, 14].

Emerging evidence indicates that epigenetic mechanisms controlling histone acetylation are dysregulated in several experimental models of cardiovascular diseases including heart failure, hypertension, aortic aneurism, and diabetes [15–19]. Hitherto, the role of histone acetylation-related mechanisms in atherosclerosis [20] and, in particular, in the regulation of Nox5 expression is poorly defined.

Histone acetylation is catalyzed by specialized enzymes belonging to the histone acetyltransferase (HAT) superfamily comprising type A HAT (e.g., p300/CBP, GNAT, MYST, and NRCF) that are typically localized in the cell nucleus and type B HAT (e.g., HAT1, HAT2, HAT4, HatB3.1, and Rtt109) that can shuttle between the nucleus and the cytoplasm. HAT-mediated lysine acetylation of the amino-terminal tails of nucleosomal histones induces chromatin relaxation, a condition that enables transcription factor—DNA interactions, and consequently the activation of gene transcription [15]. Interestingly, HAT isoenzymes control gene expression by acting on several transcription factors with an important role in cardiovascular pathology, including NF-*κ*B, hypoxia-inducible factor- (HIF-) 1, SMAD, and cAMP response element-binding protein (CREB) [21].

Considering the critical role of Mac and Nox5-derived ROS in atherosclerosis, we designed experiments to search for the existence of mechanistic links between HAT system and Nox5 overexpression in Mac in atherosclerosis. To this purpose, human nonatherosclerotic and atherosclerotic arterial specimens and cultured THP-1 monocyte- (Mon-) derived Mac were examined and tested.

Toll-like receptors (TLR) are known to play an important role in Mac activation and function in atherosclerosis [22]. Thus, to investigate the role of histone acetylation-based epigenetic mechanisms on the proinflammatory signalling-induced Nox5 upregulation in human Mac, the cells were exposed to Gram-negative bacterial lipopolysaccharide (LPS), a typical TLR4 ligand [22]. We provide evidence that in human atherosclerotic plaques, upregulated p300, HAT1, H3K27ac, H3K9ac, and Nox5 proteins are localized in the area of lesional Mac. Cultured LPS-activated Mac exhibited a dose-dependent upregulation of HAT1, H3K27ac, H3K9ac, and Nox5. In these cells, pharmacological inhibition of HAT significantly reduced the LPS-augmented Nox5 gene and protein expression levels. These data highlight the existence of a novel regulatory HAT-Nox5 mechanism that could contribute to ROS overproduction in Mac during inflammation-associated atherosclerosis.

## 2. Materials and Methods

### 2.1. Materials

If not otherwise indicated, cell culture, reagents, and kits for biochemistry and molecular biology were obtained from Sigma-Aldrich, Thermo Fisher Scientific, Qiagen, and Santa Cruz Biotechnology. Primary and secondary antibodies were from Santa Cruz Biotechnology, Diagenode, and Thermo Fisher Scientific/Invitrogen. Immunohistochemistry kits and reagents were obtained from Vector Laboratories. Human p300 and HAT1 expression vectors were from Dharmacon.

### 2.2. Tissue Samples

Human tissue samples derived either from nonatherosclerotic superior thyroid arteries (control) or from atherosclerotic plaques were obtained as discarded tissue from 15 patients undergoing carotid endarterectomy (University Hospital, Bucharest). Clinical characteristics of the patients are included in the Supplementary Material. The study was performed in agreement with the ethical directives for medical research involving human subjects (World Medical Association Declaration of Helsinki). Written informed consent was obtained from all patients. The study protocols were approved by the ethical committee at the ICBP “Nicolae Simionescu”.

### 2.3. Cell Culture and Experimental Design

Human THP-1 monocytic cell line procured from American Type Culture Collection (ATCC) was used. Mon-to-Mac differentiation was done by incubating THP-1 Mon for 3 days with 100 nmol/L phorbol 12-myristate 13-acetate (PMA) as previously described [9]. The resulting Mac were further exposed for 24 h to complete medium (resting Mac) or activated with 0.1 to 1 *μ*g/mL LPS in the absence or presence of the HAT inhibitors (CPTH2, C646). The RAW264.7 murine Mac obtained from American Tissue Culture Collection (ATCC) were employed as humanized in vitro model to investigate the regulation of human Nox5 gene promoter activity due to their higher plasmid DNA transfection efficiency as compared with THP-1 Mac.

### 2.4. Immunohistochemistry (IHC) and Immunofluorescence (IF)

Human nonatherosclerotic (control) and atherosclerotic tissue samples were fixed overnight in 4% paraformaldehyde in phosphate buffer 0.1 M, pH 7.4 at 4°C, cryoprotected by transfer in 5%, 10%, 20%, and 50% glycerol solutions in phosphate buffer 0.1 M, pH 7.4, and embedded in optimal cutting temperature (OCT) compound. Cryosections (5 *μ*m) were mounted onto Superfrost Ultra Plus™ microscope slides (Thermo Scientific) and subjected to IHC staining employing rabbit IgG, goat IgG, or mouse IgG Vectastain™ ABC kits (Vector Laboratories). The cryosections were exposed to the following primary antibodies: p300 (rabbit polyclonal, sc-584, dilution 1 : 50); HAT1 (goat polyclonal, sc-8752, dilution 1 : 50); H3K27ac (rabbit polyclonal, C15410174, dilution 1 : 500); H3K9ac (rabbit polyclonal, C15410004, dilution 1 : 500); Nox5 (rabbit polyclonal, sc-67006, dilution 1 : 50 or rabbit polyclonal, ab191010, dilution 1 : 200); CD68 (mouse monoclonal, sc-70761, dilution 1 : 50); CD45 (mouse monoclonal, sc-1178, dilution 1 : 50); or *α*SMA (mouse monoclonal, sc-32251, dilution 1 : 50). The nonspecific binding was determined by omitting the primary antibodies (No antibody (No Ab)). In IHC reactions, appropriate biotin-conjugated secondary antibodies and streptavidin-horseradish peroxidase were applied according to the manufacturer's protocols (Vector Laboratories). For IF studies, after incubation with primary antibodies, the sections were exposed to appropriate secondary antibodies, namely, Alexa Fluor™ 594 goat anti-rabbit IgG (H+L), dilution 1 : 500 (A11037), or Alexa Fluor™ 594 goat anti-mouse IgG (H+L), dilution 1 : 500 (A11032). Digital images were taken employing an inverted phase contrast fluorescence microscope (Zeiss Axio Observer D1) following counterstaining of the sections with hematoxylin (IHC) or DAPI/4′,6-diamidino-2-phenylindole (IF).

### 2.5. Evaluation of Lipid Depositions within the Atherosclerotic Lesions

The accumulation of lipids within human carotid atherosclerotic lesions was detected employing Oil Red O staining procedure as previously indicated [9]. Atherosclerotic plaque cryosections (5 *μ*m) were applied onto poly-L-lysine-coated microscope slides and stained for 15 min with Oil Red O solution (0.3% in 60% isopropyl alcohol). Sections were counterstained with hematoxylin solution, and digital images were taken using an inverted phase contrast microscope (Zeiss Axio Observer D1).

### 2.6. Quantitative Real-Time Polymerase Chain Reaction (Real-Time PCR)

Total cellular RNA was extracted from cultured cells by using a column-based purification kit (Sigma-Aldrich). First-strand cDNA synthesis was done employing M-MLV reverse transcriptase, according to the manufacturer's instruction (Thermo Scientific). The Nox1, Nox2, Nox4, and Nox5 mRNA expression levels were determined by amplification of cDNA (SYBR™ Green I chemistry, LightCycler™ 480 II thermocycler, Roche). Oligonucleotide primers common to all Nox5 transcription variants (i.e., Nox5*α*, Nox5*β*, Nox5*δ*, and Nox5*γ* mRNA splicing products) were used. The comparative C_T_ method [23] was employed to quantify the Nox1, Nox2, Nox4, and Nox5 mRNA expression using *β*-actin mRNA expression for internal normalization. The primer sequences were as follows: Nox1 (NM_013955) sense: 5′-CACAAGAAAAATCCTTGGGTCAA-3′, and antisense: 5′-GACAGCAGATTGCGACACACA-3′; Nox2 (KU178009.1) sense: 5′-TCACTTCCTCCACCAAAACC-3′, and antisense: 5′-CACCTTCTGTTGAGATCGCC-3′; Nox4 (NM_016931) sense: 5′-TGGCTGCCCATCTGGTGAATG-3′, and antisense: 5′-CAGCAGCCCTCCTGAAACATGC-3'; Nox5 (NM_024505) sense: 5′-CAGGCACCAGAAAAGAAAGCAT-3', and antisense: 5′-ATGTTGTCTTGGACACCTTCGA-3′; and *β*-actin (NM_001101) sense: S: 5′-CTGGCACCCAGCACAATG-3′, and antisense: 5′-GCCGATCCACACGGAGTACT-3′.

### 2.7. Western Blot Analysis

The tissue and cell protein extracts were prepared as previously described [24]. Briefly, nonatherosclerotic and atherosclerotic human tissue samples were washed in ice-cold phosphate-buffered saline (PBS, pH 7.4), resuspended in radioimmunoprecipitation assay (RIPA) buffer containing a protease inhibitor cocktail (Sigma), and subjected to homogenization using a bead homogenizer (Biospec). Cultured Mac were washed with PBS (pH 7.4), resuspended in 2x Laemmli's electrophoresis sample buffer, and incubated for 20 min at 95°C. Equal amounts of protein (tissue: 30 *μ*g, cells: 50 *μ*g) were run on sodium dodecyl sulfate-polyacrylamide gel electrophoresis (SDS-PAGE) and electroblotted onto nitrocellulose membranes. Primary antibodies against p300 (rabbit polyclonal, sc-584, dilution 1 : 200), HAT1 (goat polyclonal, sc-8752, dilution 1 : 200), H3K27ac (rabbit polyclonal, C15410174, dilution 1 : 1000), H3K9ac (rabbit polyclonal, C15410004, dilution 1 : 1000), Nox5 (rabbit polyclonal, sc-67006, or rabbit polyclonal, ab191010, dilution 1 : 200), and *β*-actin (mouse monoclonal, sc-47778, dilution 1 : 500) were used. The following secondary antibodies were employed: anti-rabbit IgG-HRP (sc-2370, 1 : 2000), anti-goat IgG-HRP (sc-2378, 1 : 2000), and anti-mouse IgG-HRP (sc-2031, 1 : 2000). Images of protein bands were taken with a digital detection system (ImageQuant LAS 4000, Fujifilm, Japan). Densitometric analysis was done using TotalLab™ software.

### 2.8. Transient Transfection and Luciferase Reporter Gene Assay

Luciferase reporter gene assays were performed employing Superfect™ (Qiagen) reagent as described [19]. Briefly, RAW264.7 cells were seeded at 1 × 10^5^ cells per well into 12-well tissue culture plates, twenty-four hours before transfection. Previously generated human Nox5 promoter-derived luciferase reporter gene constructs [11] were used. The optimized plasmid concentrations were 0.9 *μ*g/mL of Nox5 promoter-luciferase construct, 0.1 *μ*g/mL pSV-*β*-galactosidase expression vector (Promega), and 0.3 *μ*g/mL of empty vector and p300 (MHS1010-202699710, clone ID 6151364) or HAT1 (MHS6278-202801266, clone ID 5201102) expression vectors. The activity of the Nox5 gene promoter was determined from the ratio of firefly luciferase to *β*-galactosidase levels (Beta-Glo™ assay system, Promega).

### 2.9. Chromatin Immunoprecipitation (ChIP) Assay

ChIP and real-time PCR ChIP assays were done employing reagents and protocols from Santa Cruz Biotechnology as previously indicated [19]. The following primary antibodies (2 *μ*g/ChIP) were used for immunoprecipitation: p300 (rabbit polyclonal, sc-584), HAT1 (goat polyclonal, sc-8752), H3K27ac (rabbit polyclonal, C15410174), and H3K9ac (rabbit polyclonal, C15410004).

### 2.10. Statistical Analysis

Data obtained from at least three independent experiments were expressed as mean ± standard deviation. Statistical analysis was done by *t*-test or one-way analysis of variance followed by Tukey's post hoc test; *P* < 0.05 was considered as statistically significant.

## 3. Results

### 3.1. The Level of p300, HAT1, and H3K27ac Proteins Is Increased in Atherosclerotic Human Carotid Arteries

**Figure 1:**
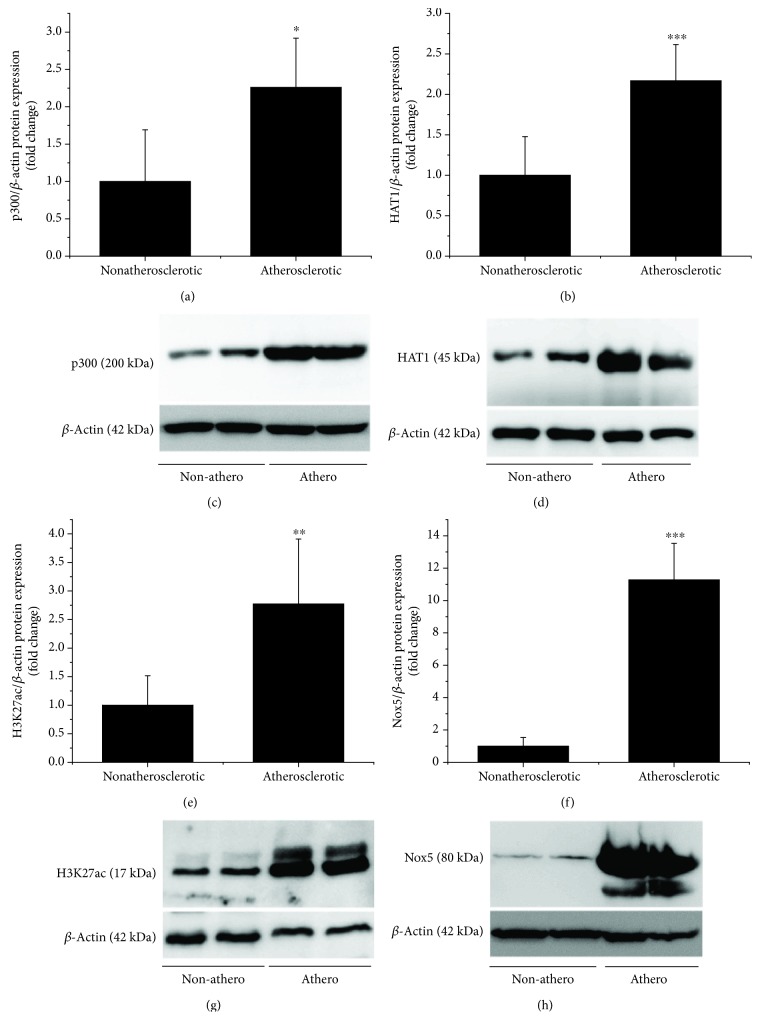
Human atherosclerotic plaques exhibit a significant upregulation of p300, HAT1, H3K27ac, and Nox5 proteins. Note the densitometric analysis of type A-HAT (p300) (a), type B-HAT (HAT1) (b), H3K27ac (e), and Nox5 (f) protein levels analyzed by western blot of atherosclerotic (carotid artery) and nonatherosclerotic (superior thyroid artery) tissues derived from patients undergoing extended endarterectomy. *n* = 8‐11; ^∗∗^*P* < 0.01, ^∗∗∗^*P* < 0.001. *P* values were taken in relation to nonatherosclerotic condition. Representative immunoblots depicting the induction of p300 (c), HAT1 (d), H3K27ac (g), and Nox5 (h) proteins in atherosclerotic carotid arteries. Non-athero: nonatherosclerotic tissue; Athero: atherosclerotic tissue.

To examine the expression pattern of key epigenetic markers of the HAT system, we used human nonatherosclerotic (superior thyroid artery) and atherosclerotic tissue samples (carotid artery). Western blot analysis revealed significant increases in p300 (≈2.25-fold) and HAT1 (≈2.16-fold) protein levels in atherosclerotic tissues above the values obtained in nonatherosclerotic specimens. Determination of the characteristic epigenetic marker of active gene expression, the H3K27ac, revealed a significant induction (≈2.77-fold) in atherosclerotic samples. The augmented p300, HAT1, and histone acetylation levels correlated with a robust upregulation (≈11.27-fold) of Nox5 subtype protein expression (Figure 1).

We have previously shown that Nox5*α* and Nox5*β* transcription variants are expressed in human Mon [9]. To examine the expression and regulation of Nox5 protein in human arterial tissues and cultured Mac, two different rabbit polyclonal antibodies raised against Nox5 protein C-terminal domain (NP_078781.3) were used, namely, anti-Nox5 antibody sc-67006 (Santa Cruz Biotechnology) and anti-Nox5 antibody ab191010 (Abcam). Both antibodies intensely labeled protein bands of ≈80-90 kDa in nonatherosclerotic (superior thyroid artery) and atherosclerotic (carotid artery) tissue samples. An additional protein band of ≈65 kDa was detected with sc-67006 antibody in protein extracts derived from atherosclerotic carotid arteries (Figure 1(h)). In accordance with the predicted molecular weight of the Nox5 protein subtypes (i.e., Nox5*α* ≈ 84 kDa, Nox5*β* ≈ 82 kDa, Nox5*δ* ≈ 85 kDa, Nox5*γ* ≈ 86 kDa, and Nox5*ε* ≈ 65 kDa), we may assume that the ≈80-90 kDa protein band matches the expected molecular weight of Nox5*α*/*β* whereas the ≈65 kDa protein band corresponds to Nox5*ε*.

**Figure 2:**
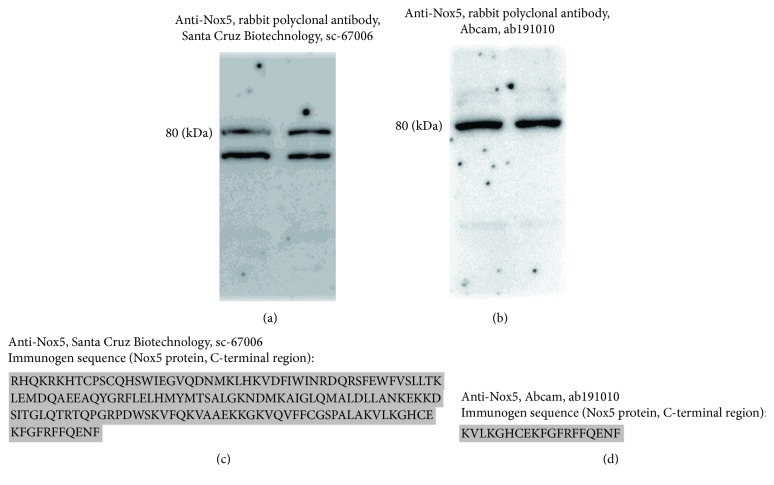
Comparative reactivity analysis of two different anti-Nox5 antibodies in protein extracts derived from cultured human Mac. Representative immunoblots depicting the Nox5 protein expression in Mac employing sc-67006 antibody (a) or ab191010 antibody (b). Immunogen sequences of sc-67006 antibody (c) and ab191010 antibody (d).

Two protein bands of ≈80-90 kDa and ≈65 kDa were detected in cultured human Mac employing the sc-67006 antibody (Figure 2(a)). In contrast, the ab191010 antibody intensely labeled protein bands of ≈80-90 kDa (Figure 2(b)). According to the sc-67006 antibody datasheet, the ≈65 kDa band could be attributed to the Nox5*ε* isoform. However, the unspecific antibody recognition of other protein(s) due to sequence homology should be considered. Noteworthily, although both antibodies were raised against Nox5 protein C-terminal region, the immunogen sequences are substantially different (Figures 2(c) and 2(d)). Considering these important aspects, only the ≈80-90 kDa protein bands were considered for quantification in western blot assays.

### 3.2. p300, HAT1, H3K27ac, H3K9ac, and Nox5 Proteins Are Located in Mac-Rich Area within Human Carotid Atherosclerotic Lesions

**Figure 3:**
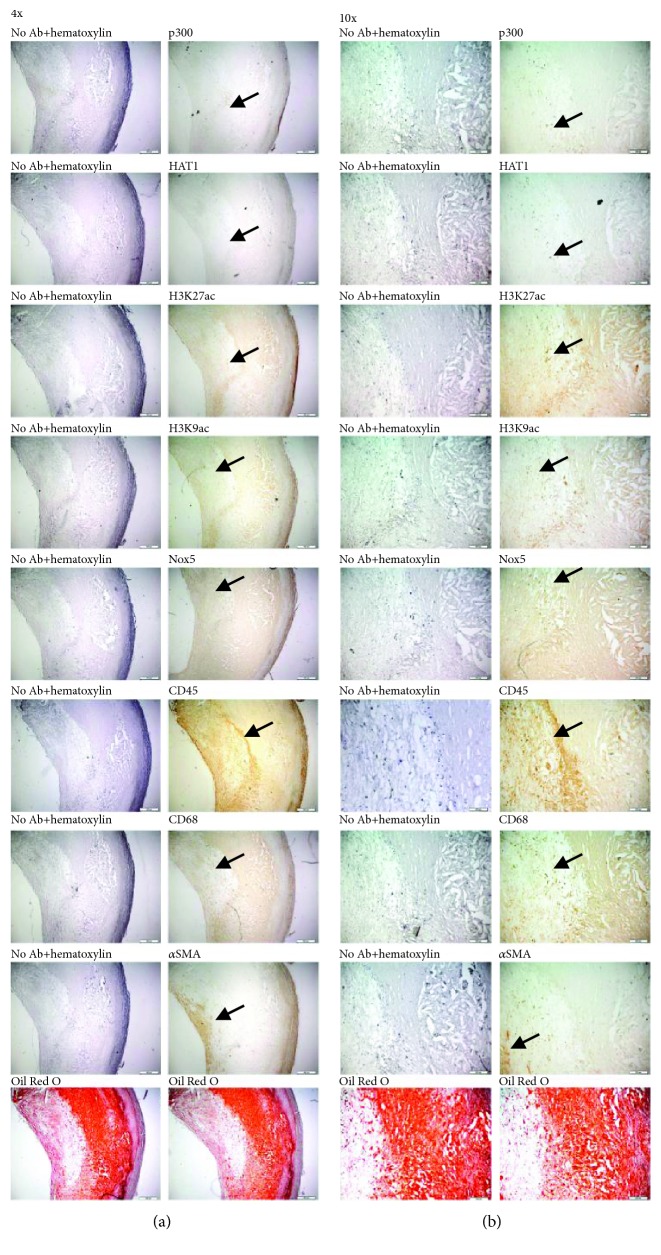
IHC localization of p300, HAT1, H3K27ac, H3K9ac, and Nox5 proteins in the area of infiltrated immune cells/Mac, fibrous cap, and lipid-rich core of a human carotid atherosclerotic plaque. The images ((a) 4x magnification, (b) 10x magnification) show representative IHC staining of 5 *μ*m thick serial sections from a human carotid atherosclerotic plaque for p300, HAT1, H3K27ac, H3K9ac, Nox5, CD45, CD68, and *α*SMA proteins. The red lesional lipid deposits were detected by Oil Red O staining. The positively stained cells are marked with arrows. Note the localization of Nox5 protein in the area of fibrous cap and lipid-rich core of atherosclerotic plaque expressing markers of infiltrated immune cells (CD45, CD68) and vascular SMCs (*α*SMA). The images are representative of 4 independent IHC assays.

**Figure 4:**
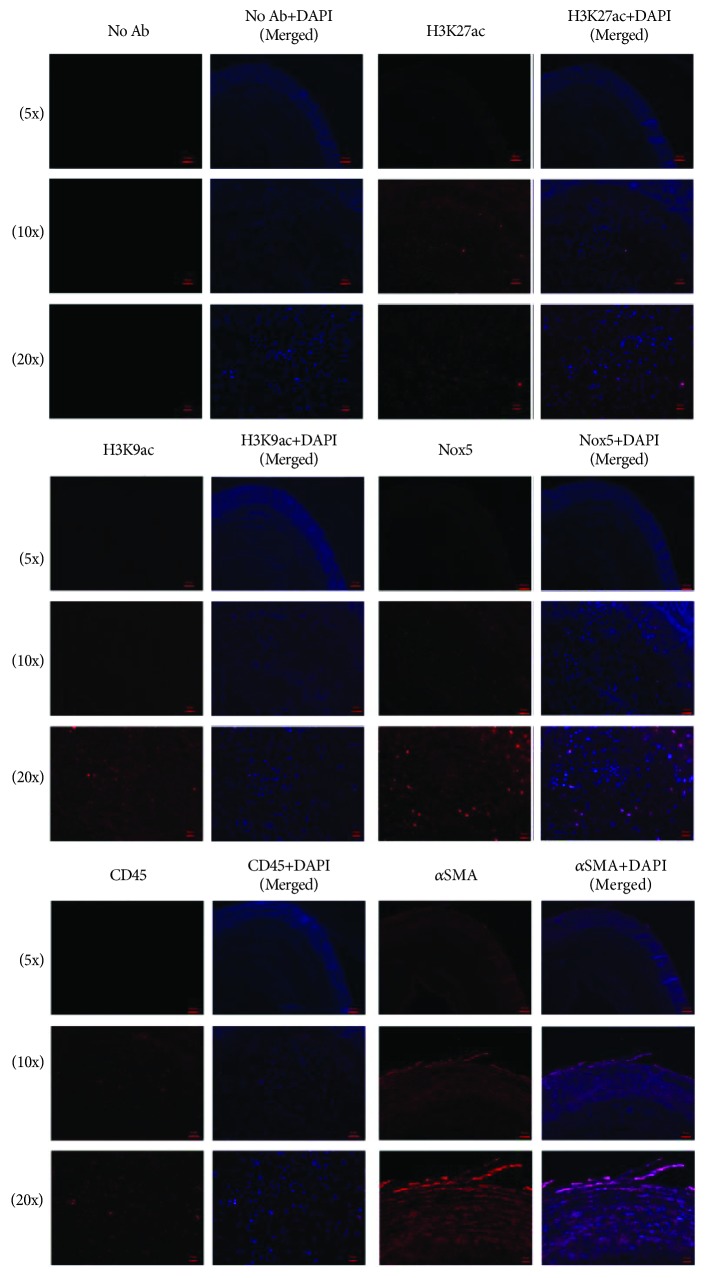
IF localization of H3K27ac, H3K9ac, and Nox5 proteins in the area of infiltrated immune cells/Mac, fibrous cap, and lipid-rich core of a human carotid atherosclerotic plaque. Representative IF images (5x, 10x, and 20x magnification) of 5 *μ*m thick adjacent frozen sections depicting positively stained cells (Alexa Fluor™ 594) for H3K27ac, H3K9ac, Nox5, CD45, and *α*SMA in a human carotid atherosclerotic plaque. Note the appearance of specific staining of proteins within fibrous cap, and lipid-rich core of atherosclerotic plaque, especially at a higher magnification (i.e., 20x).

To determine the localization of p300 and HAT1 epigenetic enzymes, histone acetylation markers (i.e., H3K27ac and H3K9ac), and Nox5 within human carotid atherosclerotic plaques, we employed IHC and IF techniques. Atherosclerotic lesion-infiltrated immune cells were identified by means of CD45 expression, a pan-leukocyte marker, and CD68, a pan-Mac marker. The immunostaining of *α* smooth muscle actin (*α*SMA) was employed for the localization of vascular smooth muscle cells (SMCs) within the fibrous cap of atherosclerotic plaque and tunica media of the carotid artery. Atherosclerotic lipid deposits were revealed employing Oil Red O staining technique as described above. IHC analysis of atherosclerotic specimens indicated that p300, HAT1, H3K27ac, H3K9ac, and Nox5 proteins are localized within the fibrous cap and lipid-rich core of atherosclerotic plaque expressing the pan-leukocyte/Mac markers (CD45 and CD68) and vascular SMC marker (*α*SMA). In negative controls, no specific IHC staining was obtained in the absence of the primary antibody (“No Ab” control) (Figure 3). Similar protein expression patterns were detected in IF studies, namely, localization of epigenetic markers of active gene expression (e.g., H3K27ac and H3K9ac) and Nox5 in the area of infiltrated immune cells/Mac in human carotid artery atherosclerotic plaques (Figure 4).

### 3.3. LPS Induces Epigenetic Alterations in Cultured Human Mac by Enhancing HAT1 Expression and Histone Acetylation

**Figure 5:**
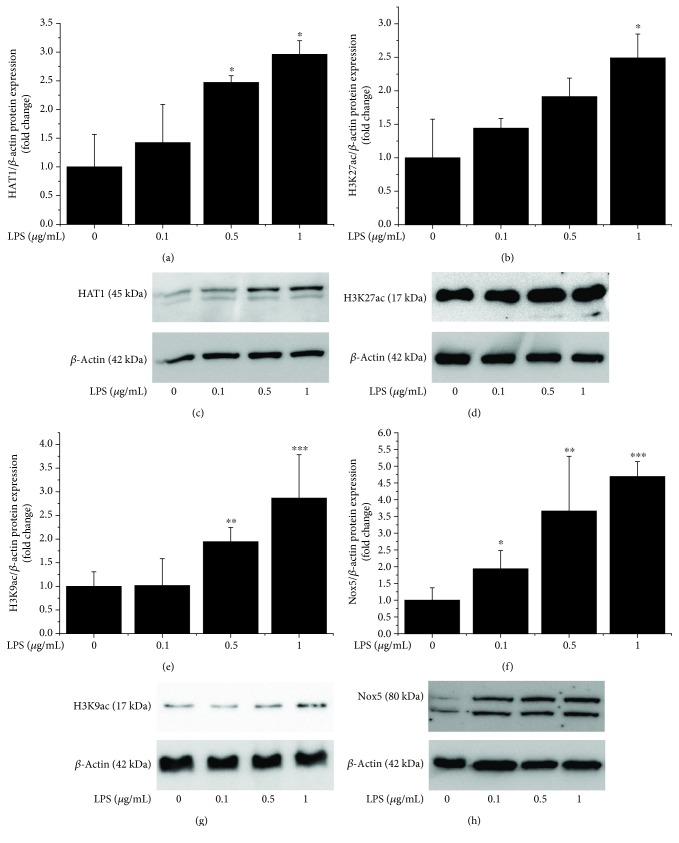
Exposure of cultured human Mac to LPS induces the upregulation of HAT1, H3K27ac, H3K9ac, and Nox5 protein levels in a concentration-dependent manner. Note the densitometric analysis of HAT1 (a), H3K27ac (b), H3K9ac (e), and Nox5 (f) protein levels analyzed by western blot in Mac exposed to increasing concentrations of LPS (0.1-1 *μ*g/mL). *n* = 3‐4; ^∗^*P* < 0.05, ^∗∗^*P* < 0.01, and ^∗∗∗^*P* < 0.001. *P* values were taken in relation to vehicle-treated cells. Representative immunoblots depicting the gradual upregulation of HAT1 (c), H3K27ac (d), H3K9ac (g), and Nox5 proteins (h) in LPS-challenged cells.

Since inflammation is instrumental in all stages of atheroma formation, we next determined the expression pattern of HAT1 and histone acetylation in cultured human Mac exposed to proinflammatory conditions. Reportedly, members of the TLR family are critical regulators of Mac activation and inflammatory function in atherosclerosis [25]. We used LPS, a common trigger of TLR4, to determine the impact of inflammatory stimuli on histone acetylation system in Mac *in vitro*. The results showed that LPS induced a steady upregulation of protein levels of HAT1 (≈3-fold at 1 *μ*g/mL), H3K27ac (≈2.5-fold at 1 *μ*g/mL), H3K9ac (≈3-fold at 1 *μ*g/mL), and Nox5 (≈4.7-fold at 1 *μ*g/mL) in cultured Mac (Figure 5). These data indicate that the activation of TLR4-dependent signalling pathways may induce transcriptomic alterations via histone acetylation-induced changes in chromatin topology and/or direct action of HAT on nonhistone proteins, such as transcription factors.

### 3.4. HAT Isoforms Mediate LPS-Induced Nox5 Gene and Protein Expression Levels in Mac

**Figure 6:**
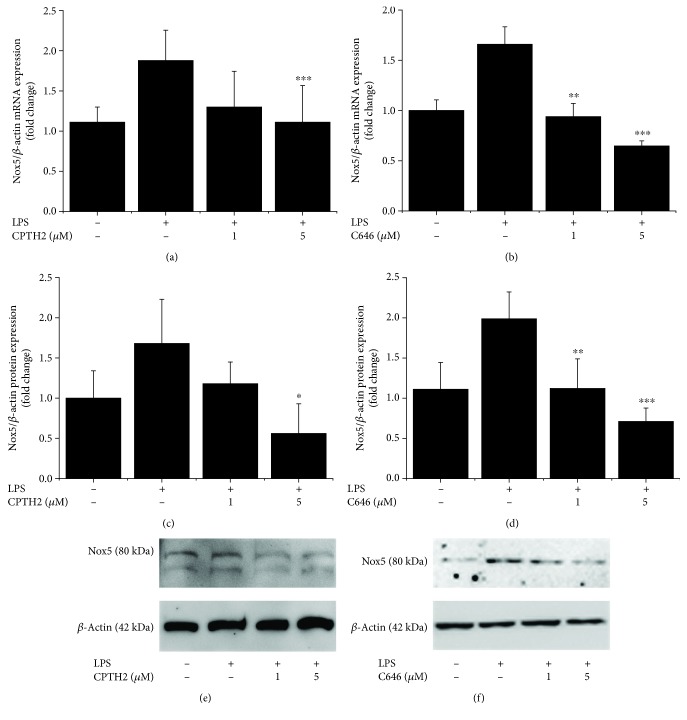
LPS-induced histone acetylation-related pathways trigger the upregulation of Nox5 gene and protein expression in Mac. Cultured human Mac were exposed for 24 h to 1 *μ*g/mL LPS in the absence or presence of CPTH2 or C646 (1-5 *μ*mol/L). The mRNA and protein levels were determined by real-time PCR and western blot. Concentration-dependent effects of CPTH2 and C646 on Nox5 mRNA (a, b) and protein (c, d) levels in LPS-exposed cells. Representative immunoblots showing the upregulation of Nox5 protein in LPS-treated cells and the regulatory effects of CPTH2 and C646 pharmacological inhibitors. *n* = 4; ^∗^*P* < 0.05, ^∗∗^*P* < 0.05, and ^∗∗∗^*P* < 0.001. *P* values were taken in relation to LPS treatment condition.

**Figure 7:**
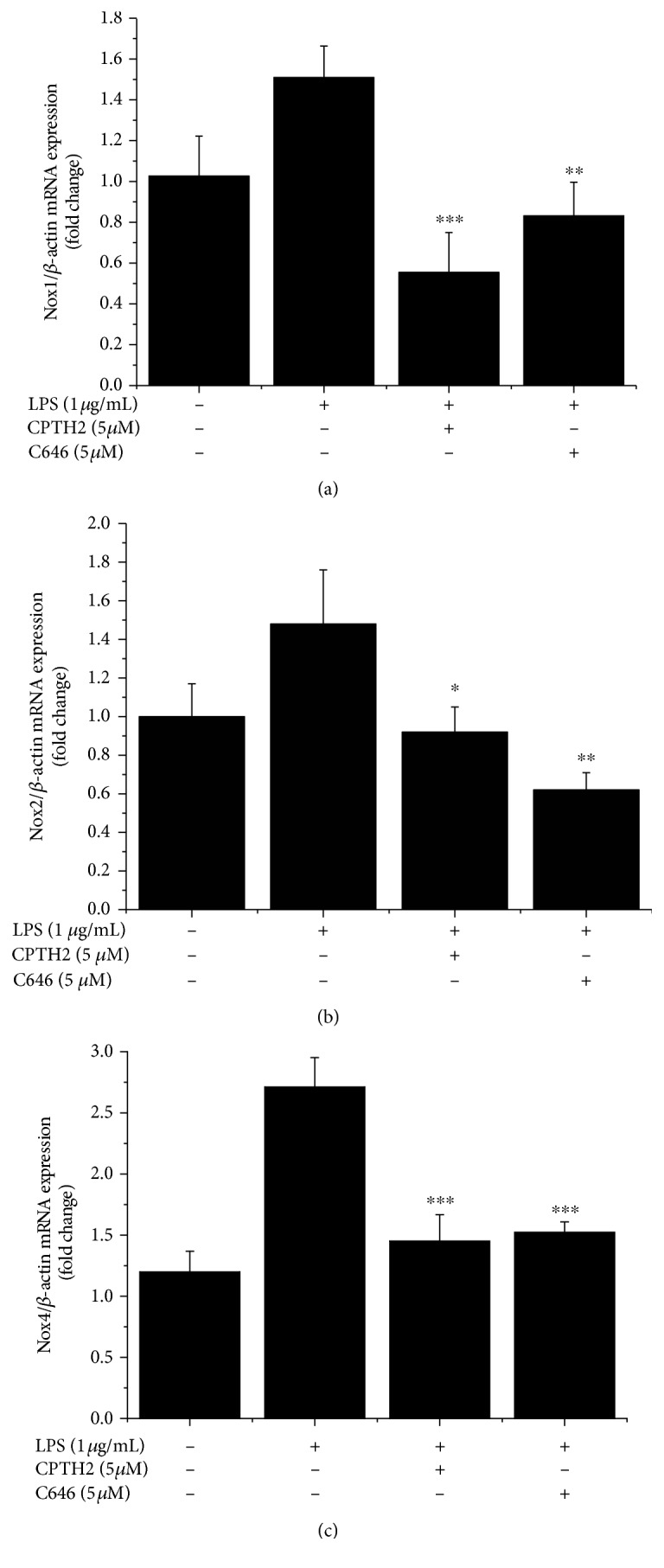
Pharmacological inhibition of HAT downregulates the mRNA expression levels of Nox1 (a), Nox2 (b), and Nox4 (c) subtypes in LPS-exposed Mac. *n* = 3; ^∗^*P* < 0.05, ^∗∗^*P* < 0.05, and ^∗∗∗^*P* < 0.001. *P* values were taken in relation to LPS treatment condition.

To further explore the existence of direct or indirect molecular mechanisms connecting inflammation-induced upregulation of HAT-related pathways and Nox5 expression in cultured human Mac, we used two highly selective, structurally different, and cell permeable pharmacological inhibitors of HAT, namely, CPTH2 {cyclopentylidene-[4-(4-chlorophenyl)thiazol-2-yl)hydrazone]} and C646 {4-[4-[[5-(4,5-dimethyl-2-nitrophenyl)-2-furanyl]methylene]-4,5-dihydro-3-methyl-5-oxo-1H-pyrazol-1-yl] benzoic acid}. CPTH2 binds to and inhibits members of the Gcn5-related N-acetyltransferase (GNAT) family whereas C646 is a specific inhibitor of p300. As shown in Figure 6, both HAT pharmacological inhibitors downregulated the LPS-augmented Nox5 mRNA and protein expression in a concentration-dependent manner. Other than Nox5, the LPS-induced mRNA expression levels on Nox1, Nox2, and Nox4 subtypes were downregulated in response to HAT inhibition (Figure 7).

### 3.5. Transient Overexpression of p300 or HAT1 Positively Regulates the Nox5 Gene Promoter Activity in Mac

**Figure 8:**
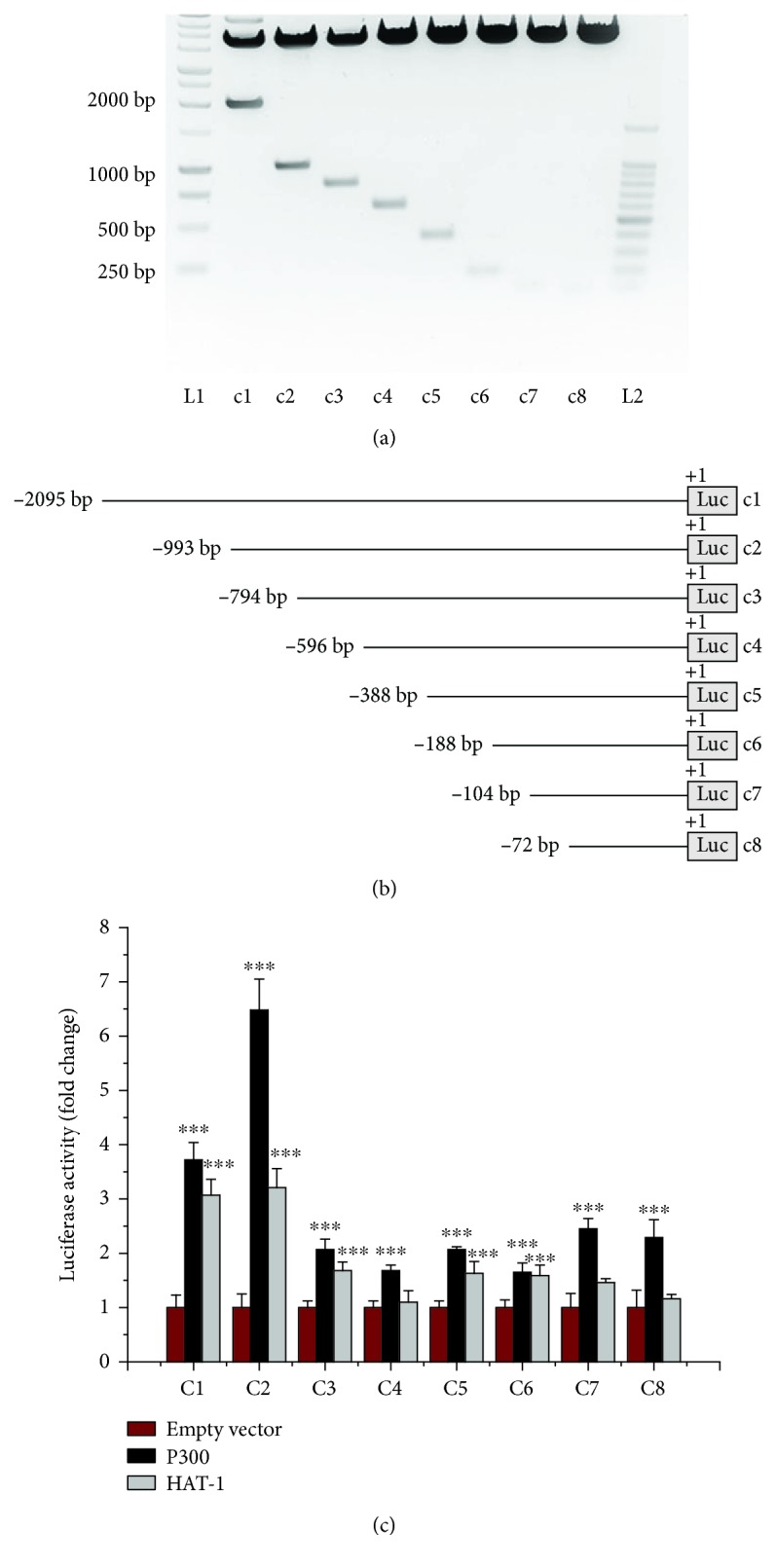
Overexpression of p300 or HAT1 upregulates the promoter activity of human Nox5 gene in Mac. The RAW264.7 cells were transiently transfected with Nox5 gene promoter-luciferase reporter gene constructs in the presence of empty vector or p300/HAT1 expression vectors. Representative agarose gel electrophoresis showing the digestion products of the c1-c8 constructs (a). Schematic depiction of the 5′-deletion mutants of the Nox5 gene promoter used in the cotransfection assays (b). Induction of luciferase level directed by the DNA regulatory elements derived from human Nox5 gene promoter in response to p300 or HAT1 overexpression in cultured Mac (c). *n* = 4; ^∗∗∗^*P* < 0.001. *P* values were taken in relation to the corresponding empty vector control.

To gain in-depth mechanistic insights on the role of p300 and HAT1 in mediating transcriptional activation of Nox5 gene in Mac *in vitro*, we performed cotransfection assays employing previously generated and characterized human Nox5 promoter-luciferase reporter gene constructs [11] and expression vectors for human p300 and HAT1. To identify the DNA regions accountable for Nox5 gene promoter activation in response to p300 or HAT1 overexpression, we used a series of Nox5 gene promoter luciferase constructs (c1 to c8) carrying the whole proximal promoter (≈2000 bp, c1) and 5′ deletion mutants (c2 to c8) generated by progressive removal of ≈200 bp DNA fragments (Figures 8(a) and 8(b)). Employing RAW264.7 murine Mac as reporter cells in transfection assays, we found that transient overexpression of p300 augmented the c1 promoter activity (≈3.7-fold) over the control level (empty vector). Interestingly, progressive 5′ deletion of ≈200 bp DNA fragments did not abolish the p300-mediated induction of luciferase level directed by the Nox5 gene promoter-derived regulatory fragments of c2 (≈6.5-fold), c3 (≈2-fold), c4 (≈1.7-fold), c5 (≈2-fold), c6 (≈1.6-fold), c7 (≈2.4-fold), and c8 (≈2.3-fold) constructs. Likewise, transient overexpression of HAT1 stimulated the transcriptional activation of the c1 (≈3-fold), c2 (≈3.2-fold), c3 (≈1.7-fold), c5 (≈1.6-fold), and c6 (≈1.6-fold) promoter elements. In contrast, the overexpression of HAT1 failed to affect the transcriptional activation of the c7 and c8 promoter regions after the removal of a larger 5′-end DNA fragments (Figure 8(c)).

### 3.6. In Human Macrophages, LPS Induces the Recruitment of p300 and HAT1 Proteins and Enhances Histone Acetylation at the Sites of Active Transcription within Proximal Promoter of Nox5 Gene

**Figure 9:**
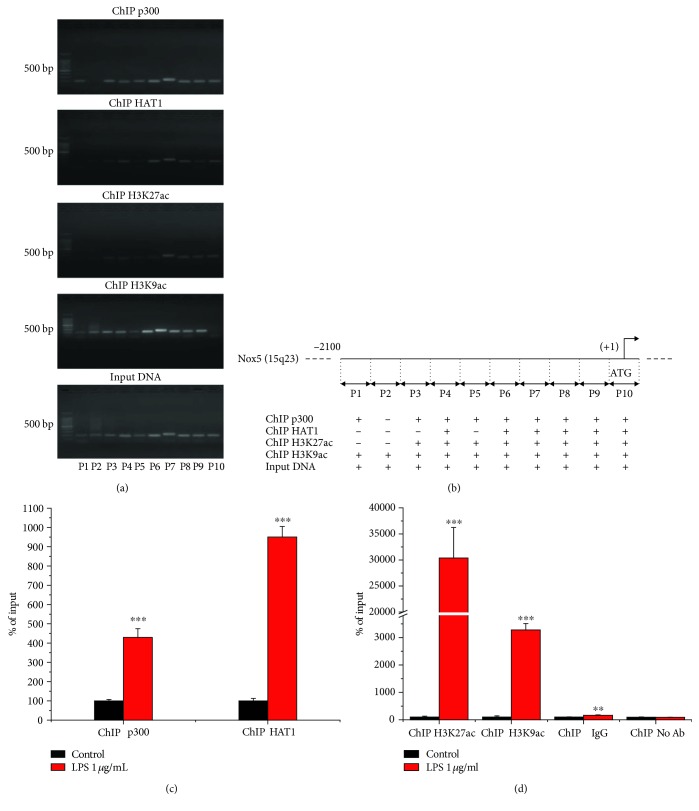
LPS induces enrichment of p300, HAT1, H3K27ac, and H3K9ac proteins at the proximal promoter region of Nox5 gene in cultured human Mac. Representative ChIP assay followed by agarose gel electrophoresis analysis of PCR products shows that p300 and HAT1 proteins are constitutively located within Nox5 gene promoter in resting Mac in vitro (a). Schematic depiction of the human Nox5 gene promoter (chromosome 15q23) showing the relative positions of the oligonucleotide primer sets spanning the entire proximal promoter region. Positive or negative DNA–p300/HAT1/H3K27ac/H3K9ac interactions are indicated with +/- marks (b). The increased abundance of p300, HAT1, H3K27ac, and H3K9ac proteins within Nox5 gene promoter in LPS-activated human Mac (c, d). *n* = 3; ^∗∗∗^*P* < 0.001. *P* values were taken in relation to resting Mac condition.

To explore whether p300- and HAT1-stimulated Nox5 transcriptional activation involves direct protein-gene promoter interactions, ChIP assays were done employing previously characterized oligonucleotide primer pairs flanking DNA binding elements of several proinflammatory transcription factors including NF-*κ*B, AP-1, STAT1/3, and C/EBP*α*/*β*/*δ* [11, 12]. ChIP assays demonstrated that p300 and HAT1 proteins were constitutively located at the sites of active transcription within Nox5 gene promoter in resting Mac, as indicated by the presence of H3K27ac and H3K9ac, important epigenetic markers of active gene expression (Figure 9(a)). The relative positions of the oligonucleotide primers used in ChIP assays, amplicon sizes, and the positive/negative protein-Nox5 gene promoter interactions are schematically depicted in Figure 9(b). The primer set P7 that generated high PCR signal in p300/HAT1/H3K27ac/H3K9ac chromatin immunoprecipitation was further used in real-time PCR-based ChIP assays aimed at quantifying the relative abundance of p300, HAT1, and acetylated histone at the level of Nox5 gene promoter in resting and LPS-activated human Mac. The results showed that compared to resting Mac, the challenging of cultured Mac with 1 *μ*g/mL LPS for 24 h resulted in significant increases in p300 (≈4.5-fold) and HAT1 (≈9-fold) abundance as well as a robust upregulation of H3K27ac (≈300-fold) and H3K9ac (≈30-fold) proteins at the proximal promoter region of human Nox5 gene. As compared with ChIP H3K27ac/H3K9ac, a slight increase (1.6-fold) in DNA fragment enrichment was detected in ChIP assays performed with normal rabbit IgG (ChIP IgG). No significant changes were detected in ChIP reactions in which the primary antibody was omitted (ChIP No Ab) (Figures 9(c) and 9(d)). Collectively, these data demonstrated that p300 and HAT1 play a role in the regulation of basic promoter activity of the Nox5 gene in resting Mac and may participate, at least in part, in the process of Nox5 upregulation in response to proinflammatory stimuli by inducing chromatin relaxation, as reported in numerous clinical and experimental models of cardiovascular diseases [11, 12, 26–28].

## 4. Discussion

Accumulating evidence indicates that besides genetic factors, epigenetic mechanisms transducing the effects of gene-(micro)environment interactions contribute to atherosclerosis by regulating the expression of key genes linked to inflammation, matrix synthesis, and cell proliferation and differentiation [29, 30]. Oxidative stress is generally acknowledged as a master regulator of numerous pathological processes leading to atheroma formation. Yet, the implication of epigenetic mechanisms in mediating ROS overproduction and oxidative stress in atherosclerosis is not entirely defined [31].

Recent studies have implicated upregulated Nox5-derived ROS in the aetiology of cardiovascular disorders (CVD), but the precise underlying mechanisms converging to Nox5 induction are not entirely elucidated [32].

Based on the fact that histone acetylation induces chromatin relaxation and activation of gene expression, we hypothesized that the upregulation of Nox5 in inflammation-associated atherosclerosis may be partially mediated by alterations in chromatin topology. To tackle this issue, we designed a systematic set of experiments on human nonatherosclerotic and atherosclerotic specimens obtained as discarded tissues from patients undergoing carotid endarterectomy; in addition, we used cultured human Mac.

The main findings of this study are (i) p300, HAT1, H3K27ac, and Nox5 protein levels are induced in human atherosclerotic carotid arteries and are abundantly expressed in the area of fibrous cap and lipid-rich core of atherosclerotic plaque expressing pan-Mac markers; (ii) exposure of cultured human Mac to LPS induces epigenetic alterations by enhancing HAT1 expression and histone acetylation (H3K27ac, H3K9ac); (iii) pharmacological inhibition of HAT reduces Nox5 gene and protein expression in LPS-challenged Mac; (iv) overexpression of p300 or HAT1 induces activation of Nox5 gene transcription; (v) the recruitment of p300 and HAT1 proteins and histone acetylation is augmented at Nox5 gene promoter in LPS-exposed human Mac.

Since Nox5 is absent in rodents, much of our current understanding of Nox5 expression, regulation, and function derives from studies on isolated tissues and cells [26, 27, 33]. Consequently, the precise implication of Nox5 in different pathologies and in particular in atherosclerosis remains elusive. Recently, it has been demonstrated that upregulated Nox5-derived ROS aggravate diabetes-induced renal failure by enhancing the expression of inflammatory molecules, excess synthesis of extracellular matrix components, and increased glomerular Mac infiltration in a human Nox5 transgenic mouse model [34, 35]. Consistent with these findings and in the absence of an inducible human Nox5 transgenic mouse model of atherosclerosis, we assume that the enhanced expression level of Nox5 and the ensuing ROS formation may contribute, at least in part, to the inflammatory and vascular remodelling reactions in the process of atheroma formation.

We have previously reported that Nox5 expression is significantly upregulated in atherosclerosis and is robustly expressed in the CD68^+^ Mac-rich area within human carotid artery atherosclerotic plaques [9]. The data of the current study further confirm and extend these observations. We provide novel evidence indicating that specific histone acetylation-regulating enzymes (e.g., p300 and HAT1) and histone acetylation (e.g., H3K27ac and H3K9ac) are induced in atherosclerotic tissues and that these epigenetic markers are largely expressed within CD68^+^ Mac- and lipid-rich regions. Our data are in good agreement with a recent study demonstrating that HAT1 mRNA level and histone acetylation are augmented in human atherosclerotic carotid arteries [36].

Human Mac in culture were used to explore the existence of direct or indirect mechanistic connections among altered histone acetylation-related pathways and upregulated Nox5 expression in response to inflammatory stimuli.

TLR control Mac activation and proinflammatory function [22, 37]. The expression levels of TLR1, TLR2, and TLR4 were found elevated in human and experimental models of atherosclerosis [38]. In addition to pathogen-derived ligands, TLR bind to and transduce the signals of oxidized low-density lipoproteins, free fatty acids, members of damage-associated molecular patterns, and necrotic cells leading to the secretion of inflammatory cytokines and chemokines that are involved in the early and advanced inflammatory phases of atherosclerotic lesion formation. Interestingly, it was shown that low-grade inflammation driven by LPS aggravates atherosclerosis in an apolipoprotein E-deficient animal model [23].

Considering these important aspects, cultured human Mac challenged with LPS were employed for in-depth molecular analysis of epigenetic regulation of Nox5 expression by histone acetylation. We found that exposure of the cells with increasing concentrations of LPS generated a steady upregulation of Nox5 expression that correlated with enhanced level of HAT1, H3K27ac, and H3K9ac proteins.

To unveil the role of HAT in mediating Nox5 upregulation in LPS-activated Mac, two different pharmacological inhibitors were used, namely, CPTH2 and C646. The former is a broad-spectrum pharmacological inhibitor of nuclear HAT isoforms belonging to the GNAT family, whereas C646 is a novel, highly potent, cell permeable, and selective inhibitor for p300 [39]. We determined that both compounds inhibited LPS-induced Nox5 mRNA and protein expression levels. Moreover, we found that the activation of HAT-dependent signalling pathways in response to LPS also contributes to the upregulation of Nox1, Nox2, and Nox4 mRNA levels in cultured Mac. These results suggest that following to LPS stimulation, chromatin undergoes conformation changes due to histone acetylation, a state that facilitates the access of proinflammatory transcription factors to their corresponding cis-acting elements in the Nox1-5 gene promoter. Notably, besides nucleosomal histones, HAT isoforms also act on and regulate the activities of numerous nonhistone proteins including transcription factors to induce or repress the gene expression. Among these, the activation of NF-*κ*B transcription factor via p300-induced acetylation of p65/NF-*κ*B subunit is the best characterized pathway leading to the activation of proinflammatory gene expression in CVD [21]. Interestingly, we have previously demonstrated that the pharmacological inhibition of NF-*κ*B reduces interferon gamma- (IFN*γ*-) augmented Nox5 mRNA and protein levels and that the transient overexpression of p65/NF-*κ*B upregulates the promoter activity of Nox5 gene in cultured human aortic SMCs [11]. Thus, we can safely assume that LPS may signal the induction of Nox5 expression in cultured Mac via two interrelated mechanisms, namely, induction of chromatin relaxation due to increased histone acetylation and NF-*κ*B activation. Other than NF-*κ*B, the function of p53 and MyoD transcription factors has been shown to be controlled by p300-mediated acetylation [40, 41].

Cotransfection assays showing that transient overexpression of p300 or HAT1 upregulates the promoter activity of human Nox5 gene also implicate nonhistone proteins (i.e., transcription factors) in the regulation of Nox5 transcription via HAT-activated molecular mechanisms. Unlike genomic DNA, plasmids carrying Nox5 promoter elements lack histones and chromatin-based control systems. Thus, the regulatory effects displayed by p300 or HAT1 overexpression are likely to be mediated by nonhistone proteins, including Nox5 gene-specific transcription factors. In addition, ChIP assays performed with primer sets spanning the entire proximal promoter region of the human Nox5 gene demonstrated that LPS-induced recruitment of p300 and HAT1 proteins is associated with elevated levels of H3K27ac and H3K9ac, important epigenetic markers of active gene expression.

Other than HAT, in a recent study we have demonstrated that pharmacological inhibition of histone deacetylase (HDAC) reduced the high glucose-induced Nox5 gene and protein expression in cultured human aortic SMCs [19]. Based on the fact that HAT and HDAC have opposite biochemical functions, it could be expected that pharmacological inhibition of these enzymes ought to induce divergent effects on Nox5 expression. Yet, despite the canonical pathway of HDAC-induced downregulation of gene expression, compelling evidence demonstrates that various HDAC inhibitors negatively regulate the expression on numerous genes linked to cardiovascular pathology including the gene coding for the Nox5 subtype [17]. Recently, it was shown that trichostatin A, a pan-HDAC inhibitor, triggers the ubiquitination and subsequent proteasomal degradation of p300 protein, a condition that impairs the formation of active transcriptional complexes at the promoter region of the Nox4 gene. By these means, both HAT and HDAC inhibitors downregulate transforming growth factor *β*1-induced Nox4 subtype expression in cultured human endothelial cells [42]. These important findings might partially explain the complex cross-communications between HAT and HDAC enzymatic systems in the process of gene regulation in both physiological and pathological conditions. Other than that, it was demonstrated that apart from p300 inhibition, C646 promotes anti-inflammatory effects by downregulating the expression of various HDAC isoforms [39].

Collectively, the data of this study strengthen the role of HAT system in mediating the induction of Nox5 expression in Mac under inflammatory conditions and provide new evidence linking dysregulated histone acetylation-related epigenetic mechanisms to ROS overproduction in atherosclerosis via Nox5 upregulation. Based on the fact that oxidative stress is instrumental in atherogenesis, pharmacological targeting of epigenetic-based pathways that control Nox5 expression might be a noteworthy novel therapeutic strategy in atherosclerosis rather than direct scavenging ROS.

## Data Availability

The data used to support the findings of this study are available from the corresponding author upon request.

## Supplementary Materials

Supplementary Materials Supplementary tables: clinical characteristics of the patients.
