# Supplementary material for: Histone Acetyltransferase-Dependent Pathways Mediate Upregulation of NADPH Oxidase 5 in Human Macrophages under Inflammatory Conditions: A Potential Mechanism of Reactive Oxygen Species Overproduction in Atherosclerosis
Source: Oxid Med Cell Longev. 2019 Sep 2;2019:3201062. doi: 10.1155/2019/3201062 (PMC6745143; doi:10.1155/2019/3201062)
Supplement: Supplementary Materials — Supplementary tables: clinical characteristics of the patients. [file 3201062.f1.pdf]

Supplementary Material

Histone acetyltransferase-dependent pathways mediate up-regulation of NADPH oxidase 5 in human macrophages under inflammatory conditions: a potential mechanism of reactive oxygen species overproduction in atherosclerosis

Mihaela-Loredana Vlad[1], Simona-Adriana Manea[1], Alexandra-Gela Lazar[1], Monica Raicu[1], Horia Muresian[2], Maya Simionescu[1], Adrian Manea[1*]

[1] Institute of Cellular Biology and Pathology "Nicolae Simionescu" of the Romanian Academy, Bucharest, Romania

[2] University Hospital Bucharest, Cardiovascular Surgery Department, Bucharest, Romania

[*]Corresponding author:

Adrian Manea, Ph.D.

e-mail: adrian.manea@icbp.ro

Institute of Cellular Biology and Pathology "Nicolae Simionescu", 8, B.P. Hasdeu Street, 050568, Bucharest, Romania

Tel: (+4021) 319 27 37

Fax: (+4021) 319 45 19

Tables: Clinical characteristics of the patients

**Parameter**	**Patient 1**	**Patient 2**	**Patient 3**	**Patient 4**
Gender: Female (F)/Male (M)	M	M	M	M
Age (years)	63	68	65	62
BMI (body-mass index) (kg/m2)	33	29	25	22
Current smoker	x	x	x	x
Former smoker	-	-	-	-
Alcohol consumption	-	-	-	-
Diabetes/duration (years)	8	-	-	-
Hypertension	x	x	x	-
Other cardiac diseases	-	-	-	-
Peripheral artery disease (PAD)	-	x	-	-
Glucose (mg/dl)	95/97/107	98	108/88	104/124
TC (mg/dl)	155	-	128	247
CHOL LDL (mg/dl)	-	178	-	170
CHOL HDL (mg/dl)	46	59	33	53
TG (mg/dl)	72	70	74	117
**Medication**				
Aspirin (mg/day)	75	75	75	-
Insulin	-	-	-	-
Metformin	x	-	-	-
Sulphonylurea	OSTERN	-	-	-
Statin	SORTIS	SORTIS	SORTIS	-

Parameter	Patient 5	Patient 6	Patient 7	Patient 8
Gender: Female (F)/Male (M)	M	F	M	F
Age (years)	73	64	57	67
BMI (body-mass index) (kg/m2)	24	23	25	-
Current smoker	-	x	x	-
Former smoker	x	-	-	-
Alcohol consumption	-	-	-	-
Diabetes/duration (years)	-	-	-	7
Hypertension	-	x	x	x
Other cardiac diseases	Coronary artery bypass grafting (CABG) x 2 Mitral valve prosthesis	-	-	-
Peripheral artery disease (PAD)	x	-	x	-
Glucose (mg/dl)	101	161/111	98/138/131	155/166
TC (mg/dl)	193	227	217	-
CHOL LDL (mg/dl)	125	132	152	-
CHOL HDL (mg/dl)	50	70	29	-
TG (mg/dl)	90	123	-	-
**Medication**				
Aspirin (mg/day)	100	75	75	75
Insulin	-	-	-	-
Metformin	-	-	-	x
Sulphonylurea	-	-	-	-
Statin	SORTIS	SORTIS	CRESTOR	CRESTOR

**Parameter**	**Patient 9**	**Patient 10**	**Patient 11**	**Patient 12**
Gender: Female (F)/Male (M)	F	M	F	M
Age (years)	43	65	67	50
BMI (body-mass index) (kg/m2)	22	25	27	26
Current smoker	x	-	-	x
Former smoker	-	x	-	-
Alcohol consumption	-	-	-	-
Diabetes/duration (years)	-	-	8	10
Hypertension	-	x	x	x
Other cardiac diseases	-	-	Mitral insufficiency	Ischemic heart disease
Peripheral artery disease (PAD)	-	-	-	x
Glucose (mg/dl)	88/98	184/136	181/117/79/133	291/241
TC (mg/dl)	127	98	152	248
CHOL LDL (mg/dl)	76	-	-	138
CHOL HDL (mg/dl)	39	30	43	47
TG (mg/dl)	(VLDL = 11,2)	123	178	(VLDL = 62)
**Medication**				
Aspirin (mg/day)	-	75	75	75
Insulin	-	-	-	x
Metformin	-	-	GLIBOMET	-
Sulphonylurea	-	-	-	-
Statin	SORTIS	CRESTOR	SORTIS	ROSUCARD

Parameter	Patient 13	Patient 14	Patient 15
Gender: Female (F)/Male (M)	M	M	M
Age (years)	73	71	75
BMI (kg/m2)	18	28	-
Current smoker	x	x	-
Former smoker	-	-	-
Alcohol consumption	x	-	-
Diabetes/duration (years)	-	12	-
Hypertension	x	-	x
Other cardiac diseases	-	Coronary artery disease	Coronary artery disease
Peripheral artery disease (PAD)	x	-	-
Glucose (mg/dl)	80/103	105/79	135/107/115
TC (mg/dl)	108	153	122
CHOL LDL (mg/dl)	55	95	92
CHOL HDL (mg/dl)	32	35	28
TG (mg/dl)	101 (VLDL=20)	111	-
**Medication**			
Aspirin (mg/day)	75	75	75
Insulin	-	-	-
Metformin	-	SIOFOR	-
Sulphonylurea	-	-	-
Statin	SORTIS	SORTIS	SORTIS

BMI: body-mass index; **TC**: total cholesterol; **CHOL LDL**: cholesterol low-density lipoprotein fraction; **CHOL HDL**: cholesterol high-density lipoprotein fraction; **VLDL:** very low density lipoprotein fraction; **TG**: triglycerides.
